# Evaluation of an Audio-Visual Novela to Improve COVID-19 Knowledge and Safe Practices Among Spanish-Speaking Individuals with Schizophrenia

**DOI:** 10.1007/s10903-023-01456-7

**Published:** 2023-02-04

**Authors:** Alex Kopelowicz, Steven R. Lopez, Gregory B. Molina, Melvin Baron, Richard Franco, Doe Mayer

**Affiliations:** 1 Department of Psychiatry and Biobehavioral Sciences, David Geffen School of Medicine at UCLA, Los Angeles, CA USA; 2 Department of Psychology, University of Southern California, Los Angeles, CA USA; 3 School of Pharmacy, University of Southern California, Los Angeles, CA USA; 4 School of Cinematic Arts, University of Southern California, Los Angeles, CA USA; 5 Olive View-UCLA Medical Center, 14445 Olive View Drive, Cottage H-2, Sylmar, CA 91342 USA

**Keywords:** Schizophrenia, Latinx, Audio-visual novela, COVID-19, Health education

## Abstract

In the United States, the health and economic consequences of the COVID-19 pandemic have disproportionately affected the Latinx community. Within the Latinx community, people with schizophrenia-spectrum disorders are more susceptible to exposure to the virus. Given their increased risk of contracting and getting sick from the virus, efforts targeting the Latinx population should focus on increasing knowledge and safe practices associated with COVID-19. We developed a 10 min animated, Spanish-language audio-visual novela designed to improve knowledge, attitudes, and behaviors regarding COVID-19. Latinx adults with schizophrenia (N = 100) at a community mental health center in Los Angeles were randomly assigned to watch the novela or a non-COVID video (control group). Participants completed surveys immediately before and one month after viewing the material. One month after watching the audio-visual novela, subjects endorsed a greater likelihood of seeking a COVID-19 vaccine than control subjects. No other significant differences were observed between the two conditions. The findings of this study suggest that the presentation of health information in a relevant, engaging, and appealing manner may be useful way to improving salutary health behaviors of Latinx people with schizophrenia-spectrum disorders.

## Introduction

### Background

The health and economic consequences of the COVID-19 pandemic have disproportionately affected the Latinx community. Across many states, their rates of contracting the virus are twice their population proportion [1]. During the height of the pandemic in Los Angeles County, the rates of hospitalization and mortality among Latinx individuals were four times higher than non-Hispanic whites [2].

Within the Latinx community, those with schizophrenia-spectrum disorders may be more susceptible to virus exposure due to cognitive impairment [3], lower awareness of risk [4], and barriers to adequate infection control including congregate living [5]. Several large studies [6–10] and a systematic review and meta-analysis [11] have demonstrated a three-fold increased risk of COVID-19 mortality in individuals with schizophrenia-spectrum disorder, even after adjusting for demographic and medical risk factors.

Given their increased risk of contracting and becoming ill from the virus, efforts targeted to the Latinx population should focus on increasing knowledge and safe practices associated with COVID-19. Moreover, this information must be presented in a format that can overcome the working memory deficits that are prominent in patients with schizophrenia [12]. Systematic reviews on the effectiveness of skills training approaches for people with schizophrenia have shown that visual narratives improve the retention of health-related information over forms of communication that rely strictly on written language (e.g., [13, 14]).

### Conceptual Framework

A commonly used form of visual narrative is the *fotonovela*, which is a small booklet that portrays a dramatic story using photographs and captions, thereby engaging the target audience with realistic characters, simple texts, and vivid images. *Fotonovelas* are designed as “Entertainment-Education,” a communication strategy that aims to educate the public on social issues through a custom-tailored piece of entertainment [15]. By presenting health education information in *fotonovela* form, viewers are immersed in the dramatic elements of the story and are more likely to internalize the messages [16]. *Fotonovelas* have been used successfully as health communication and education tools in both the United States and Mexico for medical conditions including diabetes, depression, asthma, and HIV [16–20].

We developed a 12 min, Spanish-language, digitally animated audio-visual *novela* designed to assist individuals with schizophrenia-spectrum disorders navigate the “new normal” of COVID-19. The audio-visual novela includes sound recorded by voice actors in addition to the written captions to overcome possible cognitive deficits in our patient population. In addition, we substituted illustrations for photographs of actors as we did not want to expose the production cast and crew to COVID-19. The present study describes a longitudinal, randomized control trial of the effect of the COVID-19 audio-visual novela on knowledge, attitudes, and behaviors among Latinx patients receiving outpatient mental health services at a community mental health center in Los Angeles. We hypothesized that compared to control subjects, subjects exposed to the COVID-19 material would have (1) greater knowledge about COVID-19 symptoms, risk factors, and protective behaviors; (2) more realistic attitudes regarding the risk of infection and the importance of vaccination; and (3) safer behaviors including the use of masks, frequent hand washing, and maintaining adequate physical distance in public settings.

## Methods

### Participants

The participants were 100 Spanish-speaking adults with schizophrenia-spectrum disorders receiving mental health services at the San Fernando Mental Health Center, a community mental health center operated by the Los Angeles County Department of Mental Health. The inclusion criteria were the participant self-identified as Latinx and spoke Spanish fluently; was between the ages 18 and 74; had a clinical diagnosis of a schizophrenia-spectrum disorder made by a licensed clinician; would be available for both the baseline and one-month follow up sessions; and had the ability and cognitive capacity to provide fully informed consent. There were no specific exclusion criteria for this study.

### Data Collection

Subjects with chart-diagnosed schizophrenia or schizoaffective disorder were recruited through advertisements posted at the community mental health center. Interested subjects contacted a Spanish-speaking research assistant, who obtained informed consent from the subject, administered the baseline measures (demographic information and the COVID-19 assessment battery), and randomized the subject to either the experimental or control condition using a computer-based system tailored for this project. All subjects randomized to the experimental condition were shown the COVID-19 audio-visual *novela* on a 15-inch laptop computer. Subjects randomized to the control condition were shown the Spanish-language version of the *La CLAve* movie [21], a narrative of comparable length about a young woman experiencing her first psychotic episode that includes no content regarding COVID-19. The rationale for using a non-COVID video as a comparator was to control for the non-specific benefits of watching an educational video. All subjects were contacted 1 month after baseline, at which point the Spanish-speaking research assistant re-administered the COVID-19 assessments.

### Materials

**Fig. 1:**
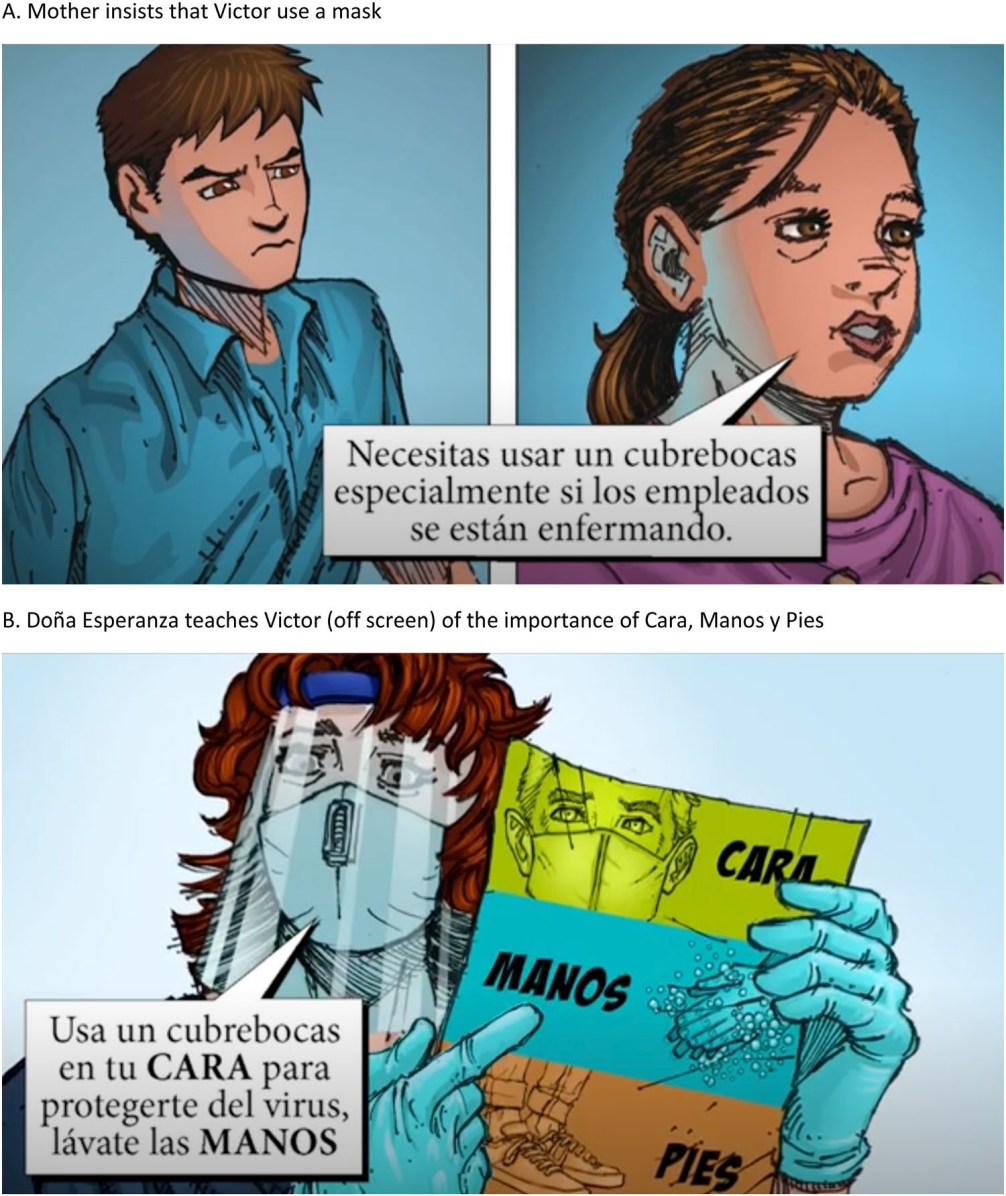
Examples of images from audio-visual novella. **A** Mother insists that Victor use a mask, **B** Doña Esperanza teaches Victor (off screen) of the importance of Cara, Manos y Pies

The audio-visual novela, entitled “*Cara, Manos y Pies*” (Face, Hands and Feet), uses computer-generated illustrations, presented frame by frame, along with oral dialogue and sound effects to tell the story of a Latinx family (a mother and her three children) coping with COVID-19. The oldest son, age 18, works in a warehouse and goes to high school but is reluctant to wear a mask and practice safe distancing. His mother, who lost her husband to a respiratory disease several years before, is worried about her son’s unwillingness to protect himself from COVID-19 infection, especially how it will impact him and his siblings. After the young man loses his job because he refuses to wear a mask at work, a family friend who is a nurse, persuades him to engage in behaviors that will decrease his risk of contracting COVID-19, including wearing a mask over his face (*Cara*), washing his hands (*Manos*) regularly, and keeping six feet (*Pies*) away from other people in public. The audio-visual novela ends with the young man agreeing to practice “*Cara, Manos y Pies*” and the entire family looking forward to the availability of a vaccine. The focus on face, hands, and feet was inspired by a local public health campaign [22]. Examples of images used in the audio-visual novela are depicted in Fig. 1. The full audio-visual *novela* is available at: https://youtu.be/J9ury0jFZuQ.

The development of the audio-visual novela was led by one of the co-authors (GM), whose team at the University of Southern California School of Pharmacy has produced numerous audio-visual novelas used throughout the world to improve health literacy among underserved populations [18–20, 23]. The script was written by Josefina Lopez, a Los Angeles-based Latinx screenwriter who has produced numerous plays and theater productions about life in urban Los Angeles. The animation was done by Luke Lizalde, a Los Angeles-based graphic artist. Several members of the research team provided feedback to the screenwriter and graphic artist during the hiring of the voice actors and other components of the production process on cultural appropriateness as well as effective communication approaches for patients with schizophrenia.

### Measures

All data was generated from interviews conducted by a Spanish-speaking research assistant. Demographic variables including age, gender, marital status, educational level, and working status were ascertained. Acculturation level was measured using the 23-item Cuellar acculturation scale [24], which assesses four content areas of acculturation: (1) language familiarity, usage, and preference; (2) ethnic identity; (3) cultural behaviors; (4) ethnic interactions. This scale yields a 5-point Likert style acculturation rating (1 = low; 5 = high). This scale has been validated with Mexican Americans and allows for continuous and dichotomous values for acculturation level.

The assessment of COVID-19 Knowledge, Attitudes, and Behaviors were derived from Spanish-language measures available through the NIH COVID-19 Survey repository at: https://www.nlm.nih.gov/dr2/COVID-19_BSSR_Research_Tools.pdf (see references [25–27]).

COVID-19 Knowledge was queried through a 12-item questionnaire that included Yes–No questions such as: “The principal symptoms of COVID-19 are fever, fatigue, dry cough and body pain” and “People with COVID-19 are not contagious if they don’t have a fever.” Correct answers received 1 point and incorrect answers received 0 points. Knowledge was calculated as the total number of correct answers on the 12-item questionnaire. Higher scores indicate greater knowledge about COVID-19. The internal consistency for this scale was 0.70 and 0.71 for the baseline and month 1 assessments.

COVID-19 Attitudes were assessed through seven questions using a 5-point Likert style rating (impossible-possible-probable-very probable-definitely). One three-item subscale measured attitudes about infection: “How likely do you think it is that the following events will happen in light of the current COVID-19 pandemic (you will be infected, someone in your family will be infected, you will be quarantined).” Cronbach alphas were 0.67 for baseline and 0.76 for month 1 assessments. The other three-item subscale assessed attitudes toward safety behaviors: “How effective are the following actions for keeping you safe from COVID-19 (wearing a face mask, washing your hands, and maintaining physical distance).” Cronbach alphas were 0.90 and 0.89 for the two assessment periods. The final item concerned attitudes toward vaccination: “How likely are you to become vaccinated when a vaccine becomes available?” Higher scores for all attitudinal items suggest more realistic attitudes towards the effects of the COVID-19 virus and what can be done to mitigate these effects.

COVID-19 Behaviors were evaluated with five items in which a 5-point Likert style rating (not at all-somewhat-half the time-mostly-always) was applied. Respondents were asked, “Over the past week, how often have you engaged in the following behaviors: (1) worn a mask, (2) maintained six feet of physical space from others when out of your house, (3) avoided contact with people who could be high risk, (4) talked to a key relative about safe practices, and (5) washed your hands with soap and water after being in a public area.” Higher scores represent increased use of safe behaviors to prevent contracting the COVID-19 virus. The Cronbach alpha for the behavior measure was 0.87 for both baseline and month 1 assessments.

### Data Analysis

SPSS Version 27 was used to carry out the data analysis. We carried out independent t-tests and Pearson Chi-square analyses to assess whether there were significant differences in the background of the participants in the two conditions. The study hypotheses were that subjects exposed to the COVID-19 material would have: (1) greater knowledge, (2) more realistic attitudes and (3) safer behaviors than control subjects. To test these hypotheses, data were collected at baseline and 1 month later. A 2 × 2 mixed design analysis of variance was carried out in which the between-subjects factor was the intervention (audio-visual novela, La CLAve) and the within-subjects factor was time (Baseline, Month 1). We conducted these analyses separately for knowledge of COVID, beliefs about infection, beliefs about safety behavior, engagement in safety behaviors, and the likelihood of obtaining vaccination when available. Finally, we carried out exploratory regression analyses to further understand the key finding.

There were five participants who did not return for the month 1 follow-up, three in the audio-visual novela condition and two in the La CLAve condition. Those five participants were dropped from the main analyses.

This study was reviewed and approved by the UCLA Institutional Review Board and the Human Subjects Research Committee of the Los Angeles County Department of Mental Health.

## Results

### Sample

**Fig. 2:**
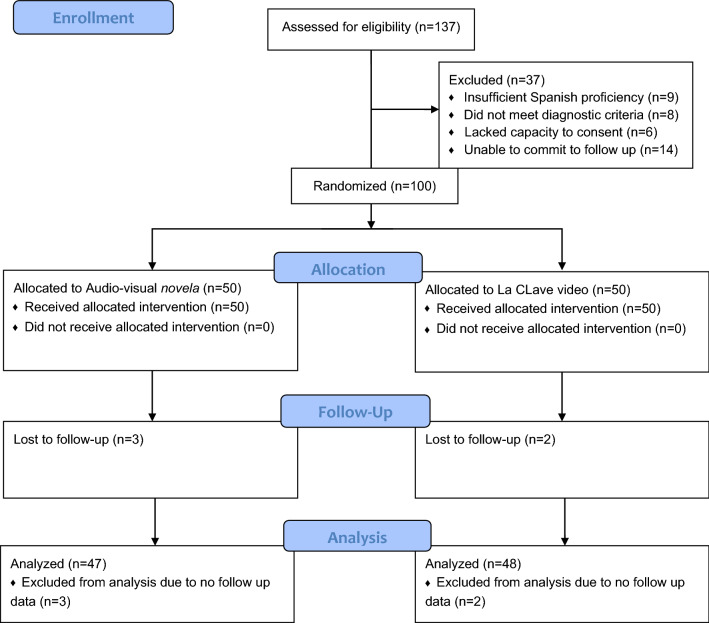
CONSORT diagram

Of the 137 people screened for eligibility, 23 did not meet inclusion criteria (insufficient Spanish language proficiency = 9; did not meet criteria for a schizophrenia-spectrum disorder = 8, lacked capacity to provide informed consent = 6). Of the 114 people invited to participate, 14 refused because they would not be available for the follow-up visit (e.g., travel plans). A total of 100 participants were consented and enrolled in the study (see Consort Diagram in Fig. 2).

The baseline characteristics of the participants are depicted in Table 1. The mean age of participants was 39.8 years (SD = 14.4, range = 20–81) and the mean level of education was 10.6 years (SD = 2.8, range = 1–16). Most participants were male (66%), never married (70%), unemployed (74%), and reported being bilingual (71%).

**Table 1:** Sample characteristics at baseline (n = 100)

Variables	Overall	Audio-visual novella (n = 50)	La CLave video (n = 50)	p-value
Demographic				
Age (in years) (mean, SD)	39.8 (14.4)	40.7 (14.3)	38.9 (14.6)	0.53
Education (in yrs) (mean, SD)	10.6 (2.8)	10.4 (3.0)	10.9 (2.5)	0.37
	n (%)	n (%)	n (%)	
Gender				0.67
Male	66 (66.0)	34 (68.0)	32 (64.0)	
Female	34 (34.0)	16 (32.0)	18 (36.0)	
Employment				0.36
Full-time	14 (14.0)	6 (6.0)	8 (8.0)	
Part time	12 (12.0)	5 (5.0)	7 (7.0)	
Unemployed	74 (74.0)	39 (39.0)	35 (35.0)	
Marital status				0.19
Never married	70 (70.0)	32 (32.0)	38 (38.0)	
Married	15 (15.0)	8 (8.0)	7 (7.0)	
Divorced	13 (13.0)	9 (9.0)	4 (4.0)	
Widowed	2 (2.0)	1 (1.0)	1 (1.0)	
Language preference				0.51
Monolingual Spanish	29 (29.0)	16 (16.0)	13 (13.0)	
Bilingual	71 (71.0)	34 (34.0)	37 (37.0)	

### Participants’ Background by Condition

There were no differences in the two conditions regarding age (p = 0.53), years of schooling (p = 0.37), gender (p = 0.67), employment status (not employed, employed, p = 0.36), marital status (never married, married at some time, p = 0.19) and language preference (Spanish or bilingual, p = 0.51).

### Intervention Effect

**Fig. 3:**
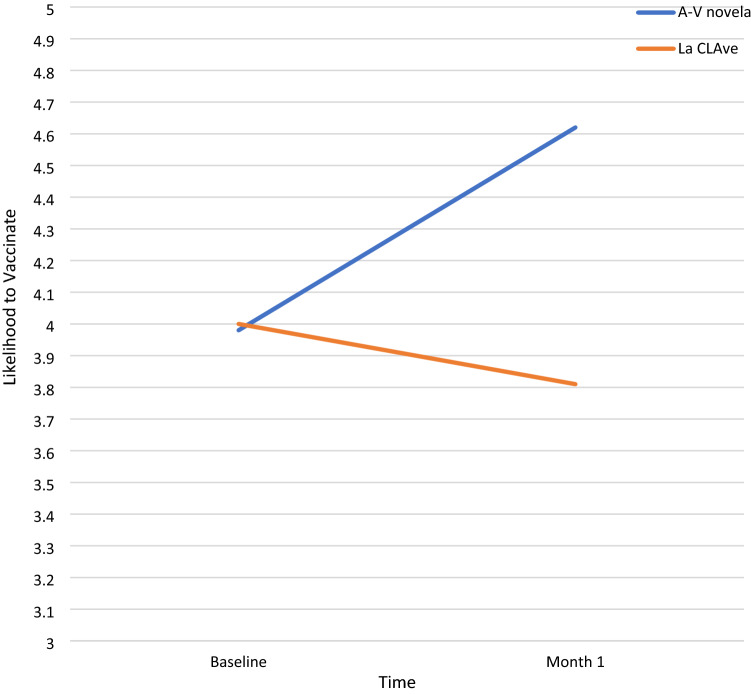
Condition × Time interaction for likelihood to seek vaccination in future

There were no statistically significant differences at baseline in the COVID measures between the two intervention groups (all ps > 0.10). We expected an interaction such that those in the audio-visual *novela* condition would show an increase from baseline to month 1 across the dependent measures, whereas the control condition would show no change. Of the five measures, only the likelihood of taking a vaccine when available showed the expected results, F(1,93) = 8.09, p = 0.005, partial η^2^ = 0.08 (see Table 2). Planned comparisons indicated that participants in the audio-visual novela condition increased their perceived likelihood of seeking a vaccine from baseline to month 1, *t*(0.46) = 3.39, p = 0.001, *d* = 0.49, whereas participants in the La CLAve condition saw no significant change over time, *t*(47) = 0.85, p = 0.40, *d* = 0.12 (see Fig. 3). The time X condition interactions for the other four measures were not significant (ps = 0.36–0.94). There were no significant video main effects (ps = 0.11–0.77). The time main effect was significant for knowledge of COVID F(1,93) = 10.44, p = 0.002, η^2^ = 0.10), attitude about safety behaviors F(1,93) = 4.97, p = 0.028, partial η^2^ = 0.05), and reported engagement in safety behaviors F(1,93) = 7.78, p = 0.006, partial η^2^ = 0.08), but not for attitude about infection (p = 0.64) or vaccination (p = 0.12). At Month 1, participants reported more knowledge of COVID (Month 1: M = 9.97, SD = 1.92; Baseline: M = 9.07, SD = 2.06), less endorsement of the effectiveness of safety behaviors (Month 1: M = 3.77, SD = 1.05; Baseline M = 4.05, SD = 1.02), and less engagement in the safety behaviors (Month 1: M = 3.49, SD = 0.0.92; Baseline: M = 3.81, SD = 0.94).

**Table 2:** Knowledge, attitudes and behavior engagement by condition and time

	Audio-visual novela				La CLAve film			
Measures	Baseline		Month 1		Baseline		Month 1	
	M	SD	M	SD	M	SD	M	SD
Knowledge	9.06	1.85	10.13	1.75	9.08	2.27	9.91	2.08
Attitudes								
Infection	2.74	1.11	2.80	1.06	2.82	0.96	2.63	1.01
Safety behavior	4.16	0.83	3.78	0.99	3.93	1.17	3.76	1.12
Vaccination^a^	3.98	1.34	4.62	1.10	4.00	1.44	3.81	1.57
Behavior engagement	3.84	0.90	3.53	0.91	3.78	0.99	3.46	0.93

Attitudes about infection and safety behavior and reported behavior engagement were each scaled (divided by their number of items) to facilitate interpretation

^a^The Condition × Time interaction was only significant for likelihood to obtain vaccination F(1,93) = 8.09, p = 0.005, partial h^2^ = 0.08. Participants in the audio-visual novela condition reported an increased likelihood of obtaining vaccination whereas participants in the La CLAve condition reported no significant change

We carried out additional analyses to explore further the increased likelihood to obtain a future vaccine for participants in the audio-visual novela condition. We first created a new variable that refers to the change in the patient’s likelihood rating of obtaining a vaccine in the future (Month 1—Baseline). A positive valence represented an increase in the likelihood judgment at month 1 and a negative valence represented a decrease in the likelihood judgment. An examination of Table 3 indicates that nearly half of all patients across both conditions (46%) reported a change in their likelihood judgment of seeking a vaccine. A chi-square analysis reveals that the distribution of change in likelihood ratings varies by condition, χ^2^(2) = 7.11, p = 0.029. There were more patients in the audio-visual *novela* condition (n = 16) who reported an increase in their likelihood ratings than in the control condition (n = 10). The audio-visual novela condition also had less patients who reported a decrease in their likelihood rating (n = 4) than in the control condition. These findings add context to the potential impact of the audio-visual novela on views about obtaining a vaccine.

**Table 3:** Changes in likelihood of seeking a vaccine over time

Condition	Decrease		No change		Increase		Total
	Frequency	Percentage	Frequency	Percentage	Frequency	Percentage	
La Clave	14	29.17%	24	50.00%	10	20.83%	48
Fotonovela	4	8.51%	27	57.45%	16	34.04%	47
Total	18	18.95%	51	53.68%	26	27.37%	95

χ^2^(2) = 7.11, p = 0.029

To identify correlates of the increased likelihood that patients in the audio-visual novela condition would seek a vaccine, we carried out a linear regression analysis with only the audio-visual novela condition. We entered the relevant baseline indicators of COVID knowledge, beliefs about infection, beliefs about safety behavior, and the actual safety behaviors that patients reported engaging in during the prior week. The outcome variable of interest was the change in the likelihood rating of getting a vaccine in the future. As can be seen in Table 4, we found that the overall model was significant (F(4,42) = 5.59, p = 0.001) accounting for 28.5% of the variance of change in the reported likelihood to vaccinate. In addition, reported knowledge of COVID was a significant independent predictor of change in likelihood to vaccinate ratings (β = − 0.38, *t* = − 2.76, p = 0.009). Given the negative valence of the beta pertaining to reported knowledge, this finding indicates that less knowledge of COVID is associated with more positive change in the reported likelihood that patients in the audio-visual novela condition would obtain the vaccine when available.

**Table 4:** Summary of linear regression analysis predicting change in vaccination likelihood in audio-visual novela condition

Variable	B	SE B	β	*p*
Knowledge	− 0.26	0.10	− 0.38	0.01
Beliefs about infection	− 0.10	0.05	− 0.27	0.05
Beliefs about safety behaviors	0.07	0.09	0.14	0.44
Engagement in safety behaviors	− 0.09	0.05	− 0.31	0.07

Adjusted R^2^ = 0.29

To ensure that the key audio-visual novela finding regarding the negative association between COVID knowledge and the likelihood to seek a vaccine was not an artifact of those whose ratings changed towards being less likely to seek vaccination, we recoded the vaccination change variable reducing it to two levels. One level included those who reported no positive change in their likelihood judgments; this includes those who reported no change in their ratings as well as those who reported a decreased likelihood of obtaining a vaccine in the future. The second level was comprised of those who reported a greater likelihood of seeking a vaccine. An independent sample t-test, indicated that those who increased their likelihood rating of seeking vaccination reported lower knowledge (n = 16, M = 7.94, SD = 1.18) than those who did not increase their likelihood rating (n = 31, M = 9.65, SD = 1.87), *t*[45] = 3.32, p = 0.002, *d* = 1.02. This is consistent with the regression findings that lesser knowledge is associated with an increased likelihood to vaccinate.

## Discussion

The purpose of this study was to test the hypotheses that Spanish-speaking individuals with schizophrenia-spectrum disorders would demonstrate increased knowledge, more realistic attitudes, and engage in safer behaviors after watching a digitally animated audio-visual novela focused on COVID-19. We found partial support for our hypotheses. Although no statistically significant differences were observed between the subjects exposed to the audio-visual novela and La CLave videos on COVID knowledge, beliefs about infection, beliefs about safety behavior, and engagement in safety behaviors, subjects who watched the audio-visual novela did report a greater likelihood of seeking a vaccine at their post-intervention assessment. This finding is consistent with several studies of *fotonovelas* [16–20], which suggest that health education messages delivered through a culturally appropriate narrative format are an effective means of changing attitudes towards health-promoting behaviors.

Contrary to our expectation that watching the audio-visual novela would increase COVID knowledge, which in turn would increase the likelihood of obtaining a vaccine, we found that subjects who reported an increased likelihood of becoming vaccinated after watching the audio-visual novela were those who were less knowledgeable about COVID. Combined with our finding that participants in both conditions demonstrated greater knowledge about COVID over time, this result suggests that the audio-visual novela delivered in a video format may be effective in positively changing attitudes towards vaccination without necessarily increasing knowledge about the illness in question. One possibility is that watching the audio-visual novela may have prompted participants to discuss the experience with a family member, sparking a dialogue that encouraged the participant to get vaccinated. We have shown that such social influences improve treatment adherence in this population [28]. Of course, it will require further study to gain a better understanding of the mechanism by which audio-visual novelas work and for whom. As an example of the former, future research should target what aspect of the characters, storyline, or message content contribute to the more positive attitude toward vaccination. In terms of the latter, it would be useful to know if audio-visual novelas, which can be widely disseminated using a variety of digital platforms, should be targeted to groups with less access to traditional health education materials.

## Limitations

These findings are subject to several limitations. The intervention itself was very brief and delivered only once. Given the cognitive deficits often found in people with schizophrenia-spectrum disorders [29], the method and pace of information delivery may have been sub-optimal to increase participants’ knowledge and behaviors regarding COVID-19. The survey questions on COVID-19 related behaviors relied on self-report rather than behavioral observation. As this study was conducted prior to the widespread availability of vaccines for COVID, we were unable to confirm if expressing an intention to vaccinate translated into actual vaccinations. Future studies should include a longer follow-up period and should measure behavioral outcomes (e.g., vaccinations). In addition, this study was conducted with stable outpatients who provided consent to participate. It is unclear if the results would generalize to patients with schizophrenia-spectrum disorders experiencing more psychiatric symptoms or to those less adherent to treatment than the current sample.

## Conclusion

The failure of health information to reach Spanish-speaking Latinx individuals with schizophrenia-spectrum disorders in a manner that is relevant, engaging, and appealing contributes to health and mental health disparities. The use of the “Cara, Manos y Pies” audio-visual novela can help reduce this communication gap because it has a narrative-based format that is contextualized to the cultural experiences of the targeted audience and can be widely disseminated across digital platforms. The findings of this study suggest that a COVID-19 digitally animated audio-visual novela informed by an entertainment-education approach may be a useful tool for improving attitudes towards vaccination within the Latinx community.
